# SARS-COV-2 infection during pregnancy, a risk factor for eclampsia or neurological manifestations of COVID-19? Case report

**DOI:** 10.1186/s12884-020-03275-2

**Published:** 2020-10-06

**Authors:** Alejandro Garcia Rodriguez, Sergio Marcos Contreras, Santiago Manuel Fernandez Manovel, Jose Miguel Marcos Vidal, Fernando Diez Buron, Camino Fernandez Fernandez, Maria del Carmen Riveira Gonzalez

**Affiliations:** 1 Department of Anaesthesiology and Critical Care, University Complex Hospital of Leon, 24071 City Leon, Spain; 2 Department of Obstetrics and Gynaecology, University Complex Hospital of Leon, 24071 City Leon, Spain; 3 Department of Neurology, University Complex Hospital of Leon, 24071 City Leon, Spain

**Keywords:** COVID-19, Case-report, Pregnancy, Preeclampsia, Neurological manifestations

## Abstract

**Background:**

There are no published cases of tonic-clonic seizures and posterior bilateral blindness during pregnancy and Severe Acute Respiratory Syndrome (SARS) Coronavirus (COV) 2 (SARS-COV-2) infection. We do not just face new and unknown manifestations, but also how different patient groups are affected by SARS-COV-2 infection, such as pregnant women. Coronavirus Disease 2019 (COVID-19), preeclampsia, eclampsia and posterior reversible leukoencephalopathy share endothelium damage and similar pathophysiology.

**Case presentation:**

A 35-year-old pregnant woman was admitted for tonic-clonic seizures and SARS-COV-2 infection. She had a normal pregnancy control and no other symptoms before tonic-clonic seizures development. After a Caesarean section (C-section) she developed high blood pressure, and we initiated antihypertensive treatment with labetalol, amlodipine and captopril. Few hours later she developed symptoms of cortical blindness that resolved in 72 h with normal brain computed tomography (CT) angiography.

**Conclusion:**

The authors conclude that SARS COV-2 infection could promote brain endothelial damage and facilitate neurological complications during pregnancy.

## Background

The vast majority of patients affected by SARS-COV-2 infection present flu-like symptoms [1, 2]. However, as the pandemic evolves, a broader, diverse and unknown spectrum of symptoms can be observed. An increasing number of patients with neurological symptoms have been reported [3–6]. These manifestations can go from mild and common symptoms such as anosmia or ageusia, to severe ones and less common such as a cerebrovascular accident [3–6]. Symptoms of extrapulmonary disease could be explained by the damage of the endothelial tissue caused by the virus [7].

With this new virus, we do not just face new and unknown manifestations, but also how different patient groups are affected by SARS-COV-2 infection, such as pregnant women. That is the reason why we present a case report of a pregnant woman infected with SARS-COV-2 who showed seizures and sudden blindness.

## Case presentation

We describe 35-year-old pregnant women with just hypothyroidism treated with thyroxin 88 μg every 24 h as personal background and optimal blood pressure control during pregnancy, in her 40 weeks and 6 days of gestation. Pregnancy has been controlled by hospital protocols with no evidence of obstetric risk factors.

With no clinical manifestations the previous days, the patient was brought to our hospital due to generalized tonic-clonic seizures at home. The first suspicion was of eclampsia, and that was why an emergency C-section was performed. The same day the diagnosis of SARS-COV-2 infection was confirmed by immunoglobulin rapid test (I-RT) and nasopharyngeal Polymerase Chain Reaction (PCR) of the virus. The only abnormal results in the blood test at arrival to the hospital were Lactic Acid Dehydrogenase (LDH) of 336 UI/I and D-Dimer of 5781 ng/ml. During her hospitalization, the blood creatinine values were within the normal range. The procedure was performed with general anaesthesia. Induction was made with propofol 200 mg and rocuronium 100 mg. After birth, we started fentanyl 300 μg and sevoflurane for maintenance of the anaesthesia without complications, completing postoperative care in the intensive care unit. After C-section, we initiated treatment with intravenous magnesium sulphate aiming levels between 4 to 6 mg/dl, she developed high blood pressure with values of Systolic pressure higher than 160 mmHg and diastolic pressure higher than 100 mmHg and we started intravenous labetalol 60 mg every hour for blood pressure control. After that, we introduced oral treatment with labetalol 100 mg every 12 h, captopril 25 mg every 12 h and amlodipine 5 mg every 12 h. It allowed lowering the dose of intravenous labetalol progressively until suspension. Twenty-four hours after C-section, the risk index Soluble Fms-Like Tyrosine kinase-1/ Placental Growth Factor (sFlt-1/PlGF) was 51.4988.

Few hours after C-section, the patient showed sudden blindness without other neurologic symptoms. Regarding to visual acuity: She could appreciate lights and shadows, being impossible with her degree of vision loss to achieve a low vision testing such as counting fingers or noticing moving objects with both eyes. Also, she had absence of the bilateral menace reflex (blink-to-threat reflex). The rest of the cranial nerves and neurological exam showed no pathological findings.

Due to these findings, a brain CT-scan and CT-angiography were performed, existing no anomalies in these tests. We notified the case to the Neurology department who suspected posterior reversible leukoencephalopathy as a primary diagnosis, although there was no nuclear magnetic resonance confirmation. Because of this suspicion, we began treatment with subcutaneous enoxaparin 40 mg every 24 h.

The vision of the patient was recovered progressively during the first 48 h and the next day she was moved to a COVID-19 hospitalization area with antithrombotic and antihypertensive treatment. Currently, she has not any neurological symptoms and normal blood pressure without treatment.

## Discussion and conclusion

The main manifestation of COVID-19 is influenza-like symptomatology but there are others such as anosmia or ageusia [1–6]. Not much has been said about different affectations in pregnant women or if this virus has any impact on the foetus. But it is important to have in mind that other coronaviruses such as SARS or Middle East Respiratory Syndrome (MERS) seems to increase complications such as preeclampsia [8].

In this case, we have a pregnant woman with positive COVID-19 I-RT and PCR that shows severe neurological symptoms. At first, the suspicion was eclampsia missing a diagnosis of preeclampsia during pregnancy, even when she had all the usual controls of pregnancy done and correct. The patient did not present any blood pressure alterations during those controls or in the ultrasound of the placenta. Also, she did not present any other suggestive clinic of preeclampsia. Twenty-four hours after C-section a risk index sFlt-1/PlGF was requested resulting high (51.4988) for diagnosis of preeclampsia in the next 4 weeks, but low for an imminent complication. The half-life of sFlt-1 is estimated in 1,4 days, while the half-life of PlGF is 3,7 [9], therefore the risk would not change. We also have the suspicion of neurological manifestations of COVID-19, where seizures are present in 0,7% of the cases [10], but she did not have any other clinical symptoms of COVID-19 such as headaches, ageusia or anosmia. Seizures are associated with previous history of cognitive impairment, older age, higher creatine Kinase levels and higher C-reactive protein [10].

SARS-COV-2 has been identified in brain endothelial cells [3]. Eclampsia, posterior reversible leukoencephalopathy and COVID-19 share a common pathophysiology affecting endothelial tissue [11, 12]. Therefore, we consider that SARS-COV-2 infection during pregnancy could increase the risk of suffering posterior reversible leukoencephalopathy or preeclampsia/eclampsia syndrome.

To conclude, we believe SARS-COV-2 infection could promote brain endothelial damage, triggering the cited neurological complications in our patient. To our knowledge, this is the first report of a patient with COVID-19 presenting preeclampsia associated with eclampsia versus posterior reversible leukoencephalopathy without alarm signs or symptoms. We consider further studies are needed to confirm that SARS-COV-2 infection is a risk factor to develop neurological complications of pregnant woman during pregnancy.

## Data Availability

Data are available on request due to privacy or other restrictions. The data that support the findings of this study are available on request from the corresponding author SMC. The data are not publicly available due to them containing information that could compromise research participant privacy/ consent.

## Abbreviations

COVID-19: Coronavirus Disease 2019
CT: Computed tomography
PCR: Polymerase chain reaction
LDH: Lactic Acid Dehydrogenase
SARS: Severe Acute Respiratory Syndrome
COV: Coronavirus
MERS: Middle East Respiratory Syndrome
I-RT: Immunoglobulin rapid test
C-section: Caesarean section
sFlt-1: Risk index Soluble Fms-Like Tyrosine kinase-1
PlGF: Placental Growth Factor

## References

[1] Zhou F, Yu T, Du R, Fan G, Liu Y, Liu Z (2020). Clinical course and risk factors for mortality of adult inpatients with COVID-19 in Wuhan, China: a retrospective cohort study. Lancet Lond Engl.

[2] Wang D, Hu B, Hu C, Zhu F, Liu X, Zhang J, et al. Clinical characteristics of 138 hospitalized patients with 2019 novel coronavirus-infected pneumonia in Wuhan, China. JAMA. 2020. 10.1001/jama.2020.1585. 10.1001/jama.2020.1585 PMC7042881 32031570

[3] Mao L, Jin H, Wang M, Hu Y, Chen S, He Q, et al. Neurologic manifestations of hospitalized patients with coronavirus disease 2019 in Wuhan, China. JAMA Neurol. 2020. 10.1001/jamaneurol.2020.1127. 10.1001/jamaneurol.2020.1127 PMC7149362 32275288

[4] Butowt R, Bilinska K (2020). SARS-CoV-2: olfaction, brain infection, and the urgent need for clinical samples allowing earlier virus detection. ACS Chem Neurosci.

[5] Giacomelli A, Pezzati L, Conti F, Bernacchia D, Siano M, Oreni L, et al. Self-reported Olfactory and Taste Disorders in Patients With Severe Acute Respiratory Coronavirus 2 Infection: A Cross-sectional Study. Clin Infect Dis. 2020. 10.1093/cid/ciaa330. 10.1093/cid/ciaa330 PMC7184514 32215618

[6] Eliezer M, Hautefort C, Hamel A-L, Verillaud B, Herman P, Houdart E, et al. Sudden and complete olfactory loss function as a possible symptom of COVID-19. JAMA Otolaryngol Neck Surg. 2020. 10.1001/jamaoto.2020.0832. 10.1001/jamaoto.2020.0832 32267483

[7] Varga Z, Flammer AJ, Steiger P, Haberecker M, Andermatt R, Zinkernagel AS (2020). Endothelial cell infection and endotheliitis in COVID-19. Lancet.

[8] Mullins E, Evans D, Viner RM, O’Brien P, Morris E (2020). Coronavirus in pregnancy and delivery: rapid review. Ultrasound Obstet Gynecol Off J Int Soc Ultrasound Obstet Gynecol.

[9] Saleh L, van den Meiracker AH, Geensen R, Kaya A, Roeters van Lennep JE, Duvekot JJ (2018). Soluble fms-like tyrosine kinase-1 and placental growth factor kinetics during and after pregnancy in women with suspected or confirmed pre-eclampsia. Ultrasound Obstet Gynecol.

[10] Romero-Sánchez CM, Díaz-Maroto I, Fernández-Díaz E, Sánchez-Larsen Á, Layos-Romero A, García-García J, et al. Neurologic manifestations in hospitalized patients with COVID-19: the ALBACOVID registry. Neurology. 2020. 10.1212/WNL.0000000000009937. 10.1212/WNL.0000000000009937 PMC7668545 32482845

[11] Mayama M, Uno K, Tano S, Yoshihara M, Ukai M, Kishigami Y (2016). Incidence of posterior reversible encephalopathy syndrome in eclamptic and patients with preeclampsia with neurologic symptoms. Am J Obstet Gynecol.

[12] Brewer J, Owens MY, Wallace K, Reeves AA, Morris R, Khan M (2013). Posterior reversible encephalopathy syndrome in 46 of 47 patients with eclampsia. Am J Obstet Gynecol.

[13] Gagnier JJ, Kienle G, Altman DG, Moher D, Sox H, Riley D (2013). The CARE guidelines: consensus-based clinical case reporting guideline development. Glob Adv Health Med.

[14] General Assembly of the World Medical Association (2014). World medical association declaration of Helsinki: ethical principles for medical research involving human subjects. J Am Coll Dent.

